# Sensitivity Analysis of an Optical Interferometric Surface Stress Ethanol Gas Sensor with a Freestanding Nanosheet

**DOI:** 10.3390/s24248055

**Published:** 2024-12-17

**Authors:** Ryusei Sogame, Yong-Joon Choi, Toshihiko Noda, Kazuaki Sawada, Kazuhiro Takahashi

**Affiliations:** 1 Department of Electrical and Electronic Information Engineering, Toyohashi University of Technology, Toyohashi 441-8580, Japan; sogame.ryusei.kr@tut.jp (R.S.); choi.yong.joon.nu@tut.jp (Y.-J.C.); noda.toshihiko.zk@tut.jp (T.N.); kazuaki.sawada@tut.jp (K.S.); 2 Institute for Research on Next-Generation Semiconductor and Sensing Science (IRES2), Toyohashi University of Technology, Toyohashi 441-8580, Japan

**Keywords:** surface stress sensor, Fabry–Perot interference, microelectromechanical systems, volatile organic compounds (VOCs), chemical sensing, ethanol

## Abstract

Ethanol (EtOH) gas detection has garnered considerable attention owing to its wide range of applications in industries such as food, pharmaceuticals, medical diagnostics, and fuel management. The development of highly sensitive EtOH-gas sensors has become a focus of research. This study proposes an optical interferometric surface stress sensor for detecting EtOH gas. The sensor incorporates a 100 nm-thick freestanding membrane of Parylene C and gas-sensitive polymethylmethacrylate (PMMA) fabricated within a microcavity on a Si substrate. The results showed that reducing the thickness of the freestanding Parylene C membrane is essential for achieving higher sensitivity. Previously, a 100-nm-thick membrane transfer onto microcavities was achieved using a surfactant-assisted release technique. However, polymerization inhibition caused by the surfactant presented challenges in forming ultrathin membranes of several tens of nanometers. In this study, we employed a surfactant-free release technique using a hydrophilic natural oxide layer to successfully form a 14-nm-thick freestanding Parylene C membrane. In contrast, the optimum thickness of the gas-adsorbed PMMA membrane was approximately 295 nm. Moreover, we demonstrated that this thinner membrane improved EtOH gas detection sensitivity by a factor of eight compared with our previously reported sensor. Thus, this study advances the field of nanoscale materials and sensor technology.

## 1. Introduction

The detection of ethanol (EtOH) gas is expected to have applications in a wide range of fields, including food, pharmaceuticals, medicine, and fuel management [1,2,3,4,5,6]. In the food industry, the quality control of marine products is crucial because the freshness of a product directly affects its market value. EtOH gas is released during the decomposition process and can be used to evaluate the freshness of marine products, poultry, pork, corn, onions, and other perishables [7,8,9]. Volatile organic compounds (VOCs) such as EtOH gas can have harmful effects on the human body, making the detection and monitoring of VOC gases essential. Moreover, VOCs are highly flammable and explosive, posing significant safety risks. Therefore, the implementation of highly sensitive sensors for monitoring VOC gases in chemical plants and similar environments is strongly desired [10,11]. IoT gas sensors, when integrated with energy harvesting technology, enable sustainable operation, offering enhanced portability and usability in environments where power supply is challenging [12,13]. Moreover, in safety monitoring of VOC gases in factories, there is an increasing demand for devices that require no battery replacement and can continuously operate even during power outages. Against this background, improving the power efficiency of sensors is essential for the development of sustainable technologies.

Various methods have been explored for sensitive detection of EtOH gas. In gas sensors using metal-oxide semiconductors, detection occurs through the interaction between EtOH gas and active ions (O^−^) on the surface of the oxide semiconductor [14]. An example of the lower detection limit and operating temperature of oxide semiconductor type sensors is shown in Table 1. Sensors using tin oxide (SnO_2_) as the gas-sensitive film have successfully detected EtOH concentrations in the range of 0.01 to 5000 ppm at 330 °C [15]. However, these sensors require the gas-sensitive SnO_2_ membrane to be maintained at high temperatures, necessitating the integration of a heater in the sensing element, which increases the power consumption. To lower the operating temperature, noble metals, such as Ag, Ti, Pd, and Pt, are often incorporated as activators and sensitizers. Nevertheless, Pt-doped SnO_2_ gas sensors still need to operate at approximately 300 °C [16]. Therefore, their operation at room temperature (24 °C) is challenging. Furthermore, in sensors using ZnO as the gas-sensitive membrane, concerns arise regarding the reduced stability owing to surface poisoning at high temperatures and degradation of fine particles in the sensing membrane. These aspects can diminish detection accuracy [17]. Other sensors using materials such as In_2_O_3_, NiO, and WO_3_ [18,19,20] also require high-temperature operations. Although some sensors have been designed to reduce power consumption by isolating the sensing component from the substrate to minimize heat transfer to the surrounding area, the inclusion of a heater complicates the manufacturing process and increases the overall footprint. Consequently, the development of gas sensors that can operate at room temperature (24 °C) without heating the gas-sensitive membrane has gained increasing attention.

EtOH gas sensors operated at room temperature include electrochemical sensors and SAW sensors. For instance, an electrochemical sensor using sulfonic acid-functionalized graphene oxide has been reported to detect EtOH gas at concentrations as low as 25 ppm [25]. However, the limitation of electrochemical gas sensors is their typical lifespan, which is usually approximately one year [26]. As an example of SAW sensors, a sensor incorporating WO_3_ nanoparticles into a polymer has been reported to detect EtOH gas down to 6 ppm [27]. Additionally, a gas sensor utilizing carbon nanofibers decorated with gold nanoparticles has been reported to detect ethanol at concentrations as low as 5 ppm [28]. However, conventional gas sensors face challenges in specific detection. As a method to enable gas type identification, surface stress sensors that facilitate arraying by selective application of gas-sensitive films are noteworthy [29]. This allows for the identification of gas types by acquiring varied response patterns from multiple gas-sensitive films.

Cantilever-based chemical sensors have been reported to operate at room temperature (24 °C) to detect surface stress changes associated with gas adsorption [30]. However, the alignment of cantilevers using laser irradiation is time-consuming and challenging to miniaturize [31]. Consequently, a membrane-type surface stress sensor (MSS) has been proposed, which, unlike conventional cantilever-based designs, features four stress-sensing regions at the fixed ends to enhance performance [31]. This approach eliminates the need for a laser-based optical system, allows measurements in solution, and is cost-effective and easily miniaturized.

Because MSS uses Si for its deformable parts, a material with a lower Young’s modulus is required to further enhance surface stress sensitivity. We proposed an optical interferometry-based micro electromechanical systems (MEMS) surface stress sensor that uses Parylene C as a freestanding membrane, which has a Young’s modulus that is two orders of magnitude lower than that of Si. This sensor, which operates based on optical interferometry, does not require a heating mechanism and can detect EtOH gas at room temperature (24 °C) by utilizing the mechanical deformation of the freestanding membrane caused by EtOH gas adsorption. In particular, a Si substrate with an air gap and a freestanding Parylene C membrane was incorporated into a Fabry–Perot interferometer. To create the freestanding Parylene C membrane, we used a surfactant-based Parylene C release technique to transfer the membrane onto a Si substrate with an air gap. In a previous study, we successfully fabricated an optical interferometric sensor using a 100 nm-thick Parylene C membrane [32]. However, to further improve the sensitivity, reducing the thickness of the freestanding Parylene C membrane is crucial. While attempting to release this membrane several tens of nanometers in size using a surfactant-based technique, the formation of pinholes and thermal stress during the transfer process split the freestanding membrane. Therefore, we have developed a surfactant-free release technique using natural oxide membranes. Although previous studies have reported devices using Parylene C membranes with thicknesses of several microns [33,34], this is the first attempt to produce a thin Parylene C membrane with a thickness of several tens of nanometers in a freestanding configuration. Surface stress sensors can enhance sensitivity to EtOH gas by thinning Parylene C, which is inherently a gas-insensitive membrane. By proposing a novel transfer process to fabricate freestanding nanometer-thick Parylene films, we achieved a lower detection limit for EtOH gas. In this paper, we present the development of a gas-sensitive membrane and report the effects of forming gas-sensitive polymethylmethacrylate (PMMA) and gas-insensitive Parylene C membranes.

In this paper, Section 2 describes the structure and detection principles of the EtOH gas sensor. Section 3 reports on the fabrication process of conventional EtOH gas sensors and discusses the challenges in fabricating Parylene C thin-film devices. Additionally, a new process that eliminates the use of surfactants is introduced. Section 4 presents the experimental setup for EtOH gas exposure tests, along with findings on time response, PMMA film thickness dependence, Parylene C film thickness dependence, and the detection limit.

## 2. Structure and Detection Principle

### 2.1. Structure and Operational Principle of the EtOH Gas Sensor

**Figure 1:**
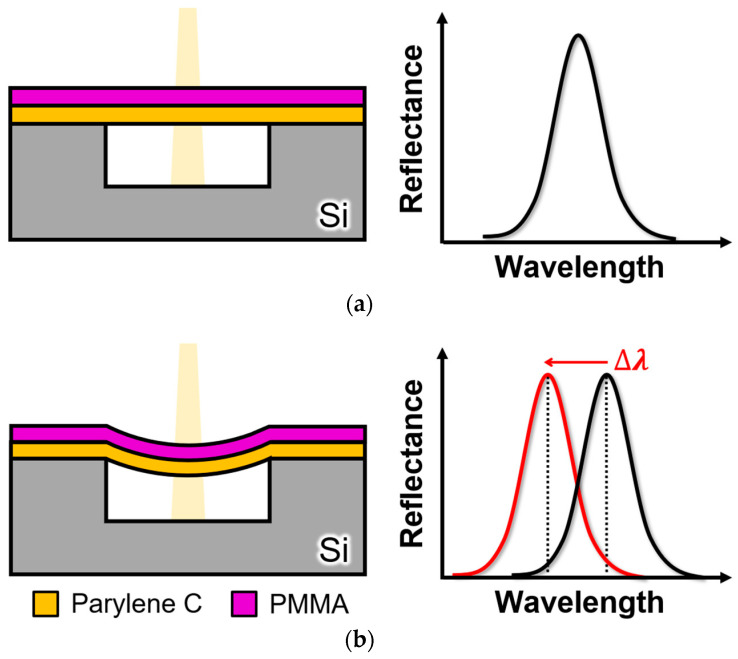
Detection principle of the proposed optical interferometric surface stress sensor for EtOH gas: (**a**) before EtOH gas exposure; (**b**) after EtOH gas exposure.

The detection principle of the proposed optical-interferometry-based MEMS surface stress EtOH gas sensor is illustrated in Figure 1. In this sensor, a microcavity is formed on a Si substrate. A bottom Parylene C layer, gas-insensitive membrane, and top PMMA layer, which shrinks upon absorption of EtOH gas, were used. These membranes were sealed to create a hollow structure, forming a Fabry–Pérot interferometer. Parylene C can be deposited using chemical vapor deposition (CVD) at room temperature, allowing precise control of film thickness on the scale of tens of nanometers. Additionally, unlike materials deposited by high-temperature CVD, it provides for the formation of flat, free-standing films free of residual stress. When EtOH gas is absorbed by the PMMA membrane, tensile stress is induced in the membrane, causing it to sink and deform. This deformation reduces the distance between the Si substrate and the freestanding membrane, shifting the peak positions of the optical interference towards shorter wavelengths. The EtOH concentration was evaluated by measuring the peak shift in the reflection spectrum. The sensor demonstrated a performance comparable to that of semiconductor-based gas sensors, even in high-humidity environments [32]. The specific selectivity for EtOH gas has not been confirmed. It has been experimentally verified that the PMMA film exhibits contraction or expansion depending on the type of gas [32]. Therefore, at present, it is difficult for a single sensor to distinguish different VOCs. However, to differentiate the response characteristics of each gas, it is expected that placing different gas-sensitive films on an array, acquiring the deformation direction and amount for each film, and applying machine learning would enable distinction between VOCs [29,35].

### 2.2. Effect of Shape Parameters on Surface Stress Sensitivity

**Figure 2:**
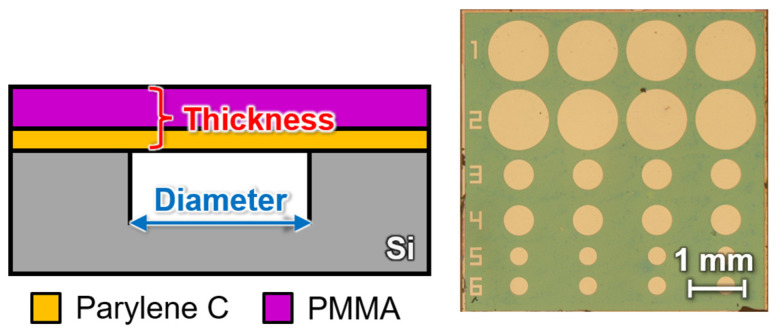
Cross-sectional structure of the proposed optical interferometric surface stress EtOH gas sensor and the actual fabricated sensor chip.

Figure 2 shows the cross-sectional structures of the proposed optical interferometric MEMS surface stress EtOH gas sensor and the fabricated one. The deformation of the freestanding membrane in this sensor is inversely proportional to the Young’s modulus and thickness of the membrane, as described by Equation (1) [36]. Therefore, reducing both the Young’s modulus and thickness of the freestanding membrane is expected to enhance sensitivity. In the proposed sensor, Parylene C is used as the freestanding membrane. In this study, we evaluated the effects of thinning both gas-sensitive PMMA membranes and gas-insensitive Parylene C membranes on the sensor sensitivity.

(1) $$\Delta z\propto \frac{D^{2}\left(1-\upsilon \right)}{Et^{2}}\Delta \sigma \;$$

## 3. Fabrication and Problem

### 3.1. Fabrication Process of the EtOH Gas Sensor

**Figure 3:**
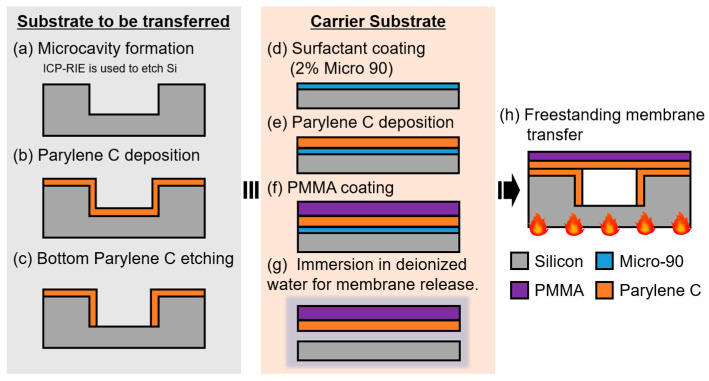
Fabrication process for the conventional optical interferometric surface stress EtOH gas sensor.

The fabrication process for conventional gas sensors is illustrated in Figure 3. A detailed description of each step is provided below.

#### 3.1.1. Microcavity Formation (Figure 3a)

The developed sensor has a hollow structure with circular microcavities. To create this hollow structure, hexamethyldisilazane (OAP; Tokyo Ohka Kogyo, Kawasaki, Japan) was spin-coated onto the Si substrate, followed by a photoresist layer (THMR-iP3100; Tokyo Ohka Kogyo). Circular patterns with diameters of 1 mm, 500 µm, and 300 µm were formed using photolithography. These patterns served as a basis for shallow Si etching of 2.5 µm, which was performed using the Bosch process in an ICP-RIE (MUC21-RD; Sumitomo Precision Products CO., LTD., Hyogo, Japan) system.

#### 3.1.2. SPM Cleaning

The SPM cleaning was performed to remove the resist. The SPM cleaning solution, prepared by mixing sulfuric acid and hydrogen peroxide in a 3:1 ratio, was used at 220 °C for 20 min, followed by rinsing with deionized water for 10 min.

#### 3.1.3. Silane Coupling Treatment

A silane coupling treatment was used to enhance the adhesion between the inorganic Si and organic Parylene C layers. The silane coupling agent was prepared by mixing deionized (DI) water, isopropyl alcohol (IPA), and γ-methacryloxypropyltrimethoxysilane (A-174) in a 100:100:1 ratio. The substrate was immersed in a silane coupling agent for 15 min and rinsed with IPA.

#### 3.1.4. Parylene C Deposition (Figure 3b)

A 100 nm-thick layer of Parylene C was deposited as a binder layer to improve the adhesion between the freestanding membrane and the Si substrate. The deposition was performed using a parylene deposition system (PDS2010; Parylene Japan, LLC., Tokyo, Japan).

#### 3.1.5. Bottom Parylene C Etching (Figure 3c)

O_2_ ashing was used to remove the Parylene C layer from the bottom surface of the cavity to eliminate the effects of optical interference within the Parylene C binder layer.

#### 3.1.6. Freestanding Membrane Preparation (Figure 3d–g)

After spin-coating a surfactant (Micro-90) onto another Si substrate, a gas-insensitive Parylene C layer was deposited using a parylene deposition system. A gas-sensitive PMMA membrane was deposited on top of the Parylene C layer using a PMMA solution (950 PMMA A11; Kayaku Advanced Materials, MA, USA) mixed with anisole.

#### 3.1.7. Freestanding Membrane Transfer (Figure 3h)

The wafer obtained in Section 3.1.6 was immersed in DI water to release the PMMA/Parylene C membrane. The released PMMA/Parylene C membrane was sealed onto the substrate with pre-formed cavities and heated at 160 °C for 1 h to ensure adhesion. Gas sensors were fabricated using this process.

### 3.2. Nanosheet Release Using Surfactants

**Figure 4:**
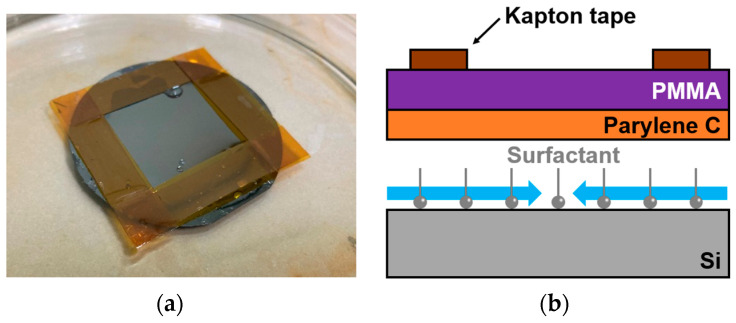
(**a**) Photograph of the nanosheet; (**b**) schematic of Parylene C nanosheet release.

In conventional methods, surfactants are used to degrade adhesion, enabling the release of PMMA/Parylene C nanosheets. Specifically, a surfactant was applied to a Si wafer, followed by the deposition of Parylene C and PMMA. By immersing the wafer in DI water, the surfactant between the wafer and the freestanding membrane was dissolved, allowing the nanosheet to peel off, as shown in Figure 4.

**Figure 5:**
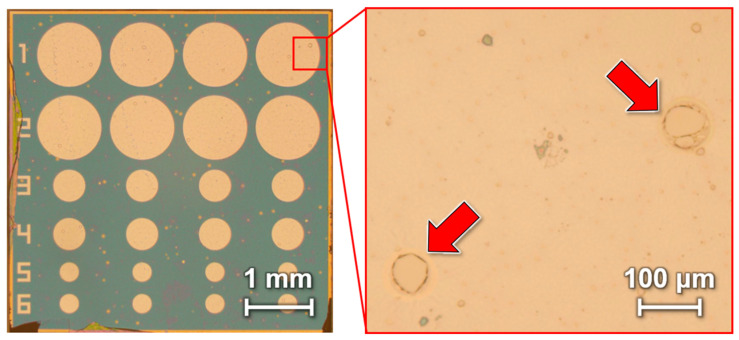
Photograph of a device with pinholes caused by thinning of Parylene-C. The red arrows indicate the pinholes.

In our previously reported gas sensor, a 200-nm-thick PMMA membrane and a 100-nm-thick Parylene C membrane were used. In this study, to evaluate the effect of a thinner Parylene C layer on the sensor performance, we fabricated a device in which the 200-nm-thick PMMA layer was maintained while the Parylene C layer was thinned to 16 nm. The device fabricated using the surfactant-release technique is shown in Figure 5. Note that pinholes were formed in the freestanding membrane. Although an EtOH gas-exposure experiment was conducted to evaluate the detection performance of the device, no deformation of the gas sensor with pinholes was observed. This is attributed to pinholes in the freestanding membrane, which likely prevent sufficient deformation of the membrane.

**Figure 6:**
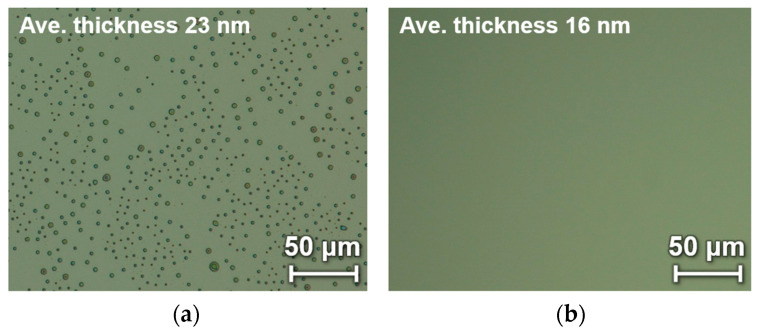
Effects of surfactants on nanometer-thick Parylene C deposition: (**a**) with surfactant coating; (**b**) without surfactant coating.

Although no pinholes were observed in the conventional 200-nm-thick PMMA/100-nm-thick Parylene C device, polymerization inhibition may have occurred when the Parylene C was thinned to a few tens of nanometers. Because surfactants are considered a possible cause of polymerization inhibition, we investigated their effect. Figure 6a shows the surface of Parylene C deposited on a Si substrate after surfactant application, whereas Figure 6b shows the surface of Parylene C deposited without surfactant. Parylene C deposition without a surfactant provided a uniform nanometer-thick thin film, whereas pinholes were observed when a surfactant was used. These results indicate that the surfactant is a major factor that inhibits the polymerization reaction during the thin-membrane deposition of Parylene C. The pinholes likely led to the formation of holes in the freestanding PMMA/Parylene C membrane during the heating process involved in the transfer.

### 3.3. New Fabrication Process Using Surfactant-Free Parylene C Release

**Figure 7:**
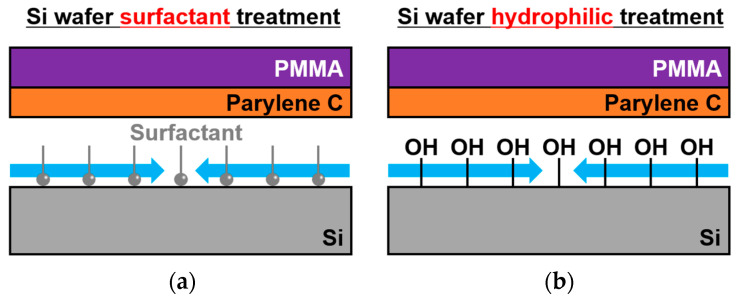
Nanosheet release process with previous method using (**a**) surfactant (Micro-90) and (**b**) proposed surfactant-free method.

**Figure 8:**
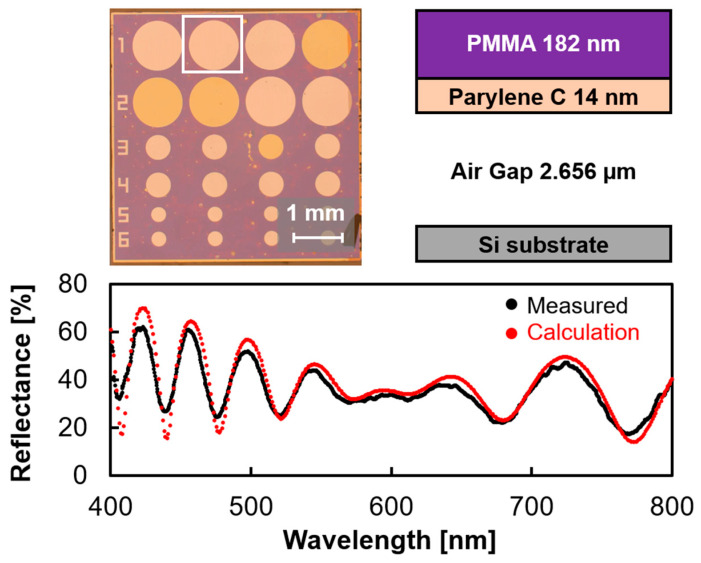
Fabricated optical interferometric gas sensor with 182 nm thick PMMA/14 nm thick Parylene C freestanding membrane using the surfactant-free process.

Figure 7 shows the surfactant-free release method using a natural oxide layer. In this method, the surface of the Si wafer is oxidized by UV-O_3_ treatment (UV-1; Samco, Kyoto, Japan) to form a hydrophilic natural oxide layer. This reduces the adhesion between the hydrophilic substrate and hydrophobic Parylene C, enabling the release of a freestanding PMMA/Parylene C membrane. The hydrophilic SiO_2_ film reduced the adhesion to the hydrophobic Parylene, allowing the fabrication of the device without inhibiting the polymerization of Parylene C. The released PMMA/Parylene C nanosheets were transferred onto a Si wafer with prepatterned cavities. Figure 8 shows the optical interferometric MEMS surface stress EtOH gas sensor fabricated using the release technique with a natural oxide layer. In contrast to the previous release methods, no defects were observed in the freestanding membranes. The reflection spectrum from the cavity area showed several interference peaks compared to the interference characteristics of the PMMA/Parylene C nanosheet, suggesting that an air gap was formed under the PMMA/Parylene C nanosheet. To calculate the air gap under the nanosheet, optical analysis was performed using the film thicknesses of PMMA (182 nm) and Parylene C (14 nm), which were measured in advance. The simulation results are in good agreement with the measured values at an air gap of 2.66 µm. Furthermore, the obtained gap value was consistent with the etching depth, indicating that the transferred film maintained a flat shape in the cavity.

## 4. Results and Discussion

### 4.1. Experimental System for Detecting EtOH Gas

**Figure 9:**
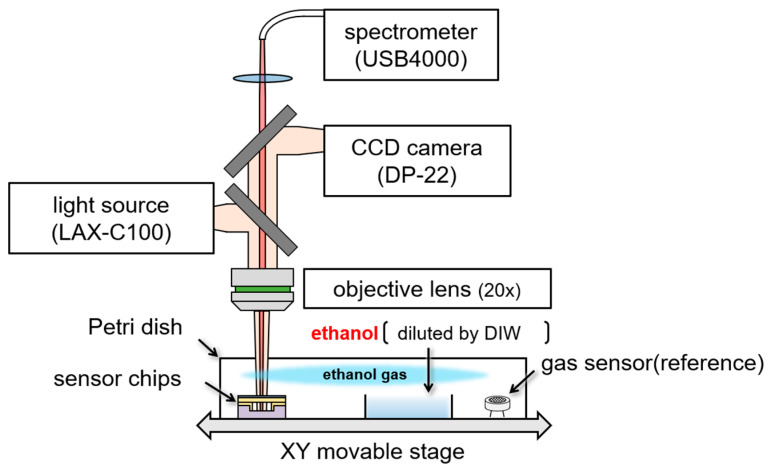
Experimental setup for measuring surface stress of gas sensors during EtOH gas exposure.

Figure 9 shows the experimental setup for measuring the surface stress of the gas sensor during exposure to EtOH gas. The sensor was mounted on a motorized XY stage. First, a Petri dish was placed over the sensor and allowed to stabilize for 3 min. Subsequently, 0.4 mL of diluted EtOH solution was introduced into the Petri dish, exposing the sensor to volatile EtOH gas for 15 min. After exposure, the Petri dish and EtOH solution were removed and the system was allowed to rest. The sensor was irradiated with light and the reflection spectra were measured using a spectrometer. The amount of membrane deformation was determined by analyzing the change in the reflection spectrum owing to the deformation of the freestanding membrane caused by EtOH gas.

### 4.2. Time Response to EtOH Gas

**Figure 10:**
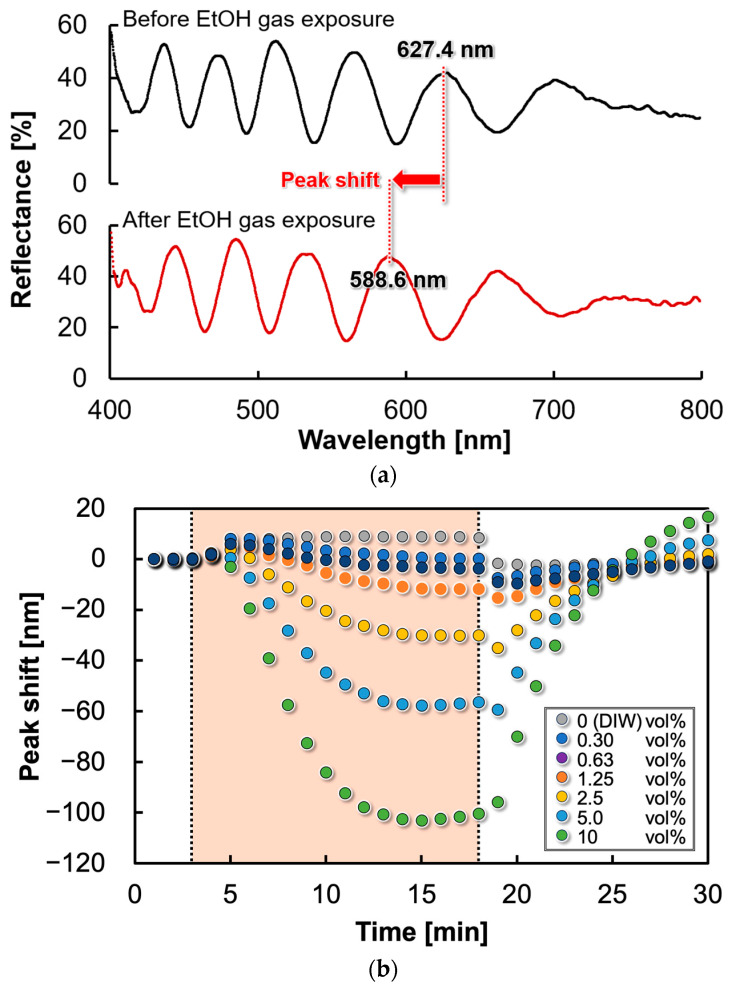
(**a**) Reflection spectra before and after EtOH gas exposure (EtOH solution concentration 2.5 vol%).; (**b**) peak shift of the reflection spectrum for each solution concentration.

Figure 10a shows the reflection spectra before and after exposure to EtOH gas. Figure 10b shows the peak shift in the reflection spectrum corresponding to EtOH concentration. The geometric parameters of the EtOH gas sensor were 1 mm diameter, 40 nm thickness of the Parylene C membrane, and 208 nm thickness of the PMMA membrane. As shown in Figure 10a, the 627.4 nm peak of the reflection spectrum before EtOH gas exposure shifted by 38.8 nm after exposure to shorter wavelengths. The downward movement of the freestanding film suggests that the contraction of the sensitive film applied tensile stress to the Parylene in the gas-insensitive film. Optical analysis using the rigorous coupled-wave analysis (RCWA) method indicated that this peak shift was due to a 125 nm displacement of the freestanding membrane. Furthermore, Figure 10b confirms that the peak shifts to shorter wavelengths with increasing EtOH gas concentration. This is attributed to the increased surface stress of the freestanding membrane owing to an increase in the concentration of the EtOH solution. For low-concentration solutions including 0 vol% (DIW), a slight redshift (film expansion) was observed after gas exposure. This is thought to be due to a response to water vapor caused by changes in humidity, as shown in previous reports [32]. After stopping the exposure to a high concentration of EtOH, the freestanding membrane deformed in the upper direction owing to the reaction force. However, it returned to its initial state.

### 4.3. Thickness Dependence of Parylene C Membrane

**Figure 11:**
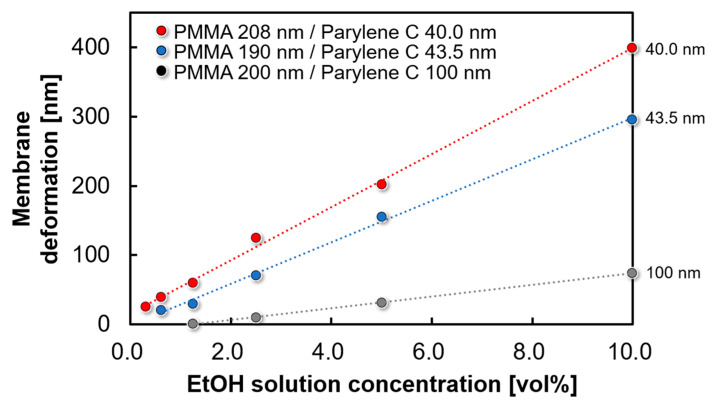
Effect of thinning the gas-insensitive Parylene C membrane on membrane deformation.

Figure 11 shows the deformation of the freestanding membrane for each EtOH solution concentration depending on the thickness of the gas-insensitive Parylene C membrane. The diameter of the EtOH gas sensor was 1 mm and the thickness of the Parylene C membrane varied from 40 nm and 43.5 nm to 100 nm. The thickness of the PMMA membrane was set to 200 nm, which is close to the experimental optimum (discussed in detail in Section 4.4), whereas the variation in the PMMA thickness between the individual membranes was less than 10%. As shown in Figure 11, as the thickness of the Parylene C membrane decreases, the deformation of the membrane increases in response to the EtOH solution concentration. Therefore, thinning the gas-insensitive Parylene C membrane is effective for improving sensor sensitivity. However, a slight effect was confirmed at a film thickness of 100 nm or less. Furthermore, deviations from the optimal value of the PMMA thickness considerably affected the sensitivity predicted from the theoretical formula.

### 4.4. Thickness Dependence of PMMA Membrane

**Figure 12:**
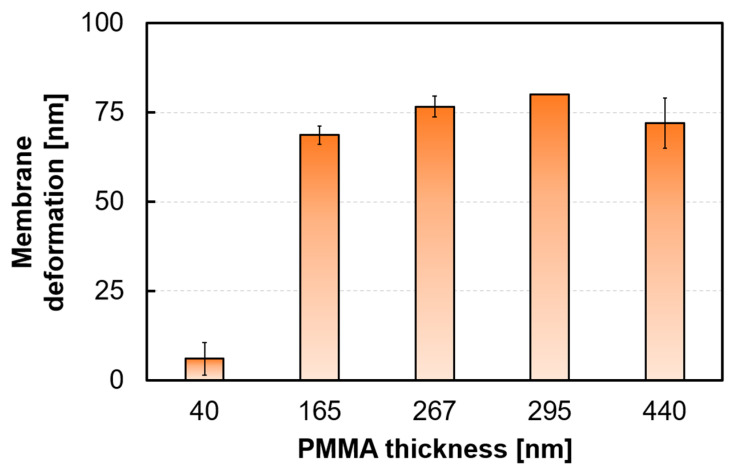
Peak shift as a function of gas-sensitive PMMA thickness.

**Figure 13:**
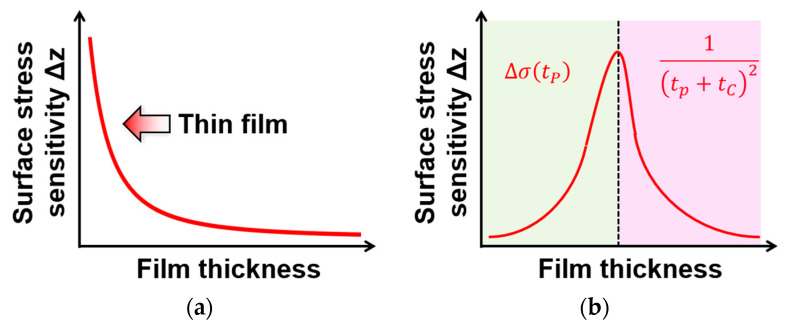
Schematic (**a**) without and (**b**) considering the dependence of the surface stress on the sensitive film thickness.

Figure 12 shows the peak shift at 10 vol% EtOH corresponding to different PMMA thicknesses. The results indicated that the peak shift of the EtOH gas sensor was the largest when the PMMA membrane thickness was approximately 295 nm. The thickness of the Parylene C membrane was set to 100 nm. Note that thinner freestanding membranes were expected to increase the sensitivity of MEMS sensors. The results confirmed that thinning the Parylene C layer improved the sensitivity. The optimal membrane thickness of PMMA can be attributed to its gas-sensitive properties. The surface stress of PMMA on Parylene C was assumed to remain constant at a particular EtOH gas concentration, even as the PMMA membrane became thinner (Figure 13a). In other words, the previous model assumed that the amount of absorbed EtOH remained constant with changes in the gas-sensitive membrane thickness at a given EtOH gas concentration. However, the experimental results suggested that the surface stress σ may depend on the gas-sensitive membrane thickness, as shown in Figure 13b. This indicates that the absorption of EtOH gas increased depending on the PMMA thickness, increasing the surface stress transmitted to the Parylene membrane. However, when the membrane thickness exceeded the optimal value, the sensitivity decreased, owing to the increased stiffness of the membrane. These results suggest that the PMMA thickness significantly influences the gas sensor sensitivity and that optimizing the membrane thickness is effective in improving sensitivity.

### 4.5. Lower Detection Limit for EtOH Gas Sensor

**Figure 14:**
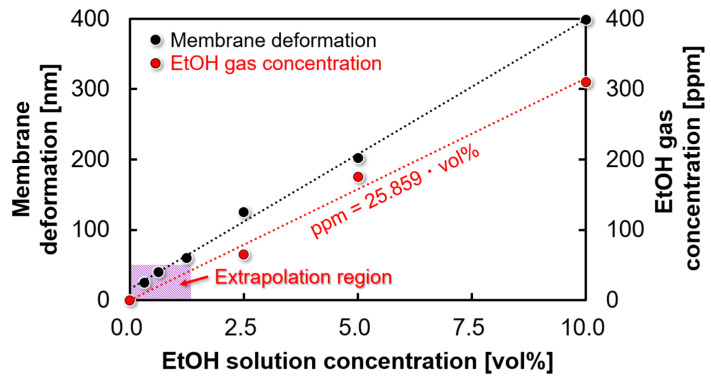
Correlation between EtOH solution and EtOH gas concentrations. The gas concentration measured by the commercialized semiconductor sensor and the deformation of the MEMS sensor are plotted on the vertical axis.

In this study, sensors with a PMMA thickness of 200 nm, Parylene C thickness of 40 nm, and diameter of 1 mm demonstrated the highest detection sensitivity. The EtOH solution used in this experiment had a lower detection limit of 0.3 vol%. A commercial semiconductor gas sensor (TGGS2620; FIGARO, Osaka, Japan) was used to investigate the correlation between the diluted EtOH solution and the EtOH gas. The semiconductor gas sensor exhibited a linear response and reliable operation at EtOH concentrations above 2.5 vol%. Figure 14 shows that the sensor successfully measured a minimum EtOH solution concentration of 0.3 vol%. The proposed sensor responded within a concentration range below the detection limit of the semiconductor gas sensor (50 ppm) used as a reference. A regression line was derived based on the linear response range of the semiconductor gas sensor. Therefore, the detection limit was calculated by extrapolation, yielding 7.76 ppm, as shown in Figure 14. Because this study and a previous report exposed the sensor to EtOH gas volatilized from an EtOH solution in the same experimental environment, the lower detection limits can be evaluated by comparing the solution concentrations. The previously reported lower detection limit for the EtOH solution concentration was 2.5 vol%. In contrast, this study successfully measured concentrations as low as 0.3 vol%, confirming ethanol gas detection at one-eighth the concentration of the previous device by adjusting the geometric parameters.

## 5. Conclusions

In this study, to fabricate a freestanding Parylene C membrane with a few tens of nanometers thickness, we developed a fabrication process to release a nanometer-thick Parylene C film by hydrophilizing the surface of the carrier substrate. We investigated the geometric parameters of an optical interferometric surface-stress sensor to improve its sensitivity. The thickness dependence of Parylene C, a gas-insensitive membrane and PMMA, a gas-sensitive membrane, was also evaluated. The results confirmed that the sensitivity increased as the Parylene C membrane was thinned. By contrast, the PMMA layer did not exhibit improved sensitivity to thinning, indicating the presence of an optimal PMMA thickness (295 nm). The developed device achieved an eightfold improvement in sensitivity compared to the previously reported device, successfully detecting EtOH solution concentrations as low as 0.3 vol% and EtOH gas concentrations up to 7.76 ppm as the lower detection limit. However, a reduction in yield associated with the thinning of the Parylene C film was also observed. Future research prospects include investigating the causes of this yield reduction due to film thinning. Through these efforts, further improvements in the detection limit can be expected.

## Figures and Tables

**Table 1:** Lower detection limit and operating temperature of oxide semiconductor type sensors.

Material	Temperature (°C)	LOD (ppm)
Highly porous SnO_2_ fibers [15]	340	0.001
Pt-doped SnO_2_ [16]	300	0.001
In_2_O_3_ porous pompon [18]	260	0.4
WO_3_ nanorods [19]	160	20
Ga-doped NiO nanoparticles [20]	250	10
Co-doped SnO_2_ nanobelts [21]	300	50
Ag-doped SnO_2_ nanoparticles [22]	180	1.0
ZnO nanorod [23]	220	1.0
ZnO nanostructured membrane [1]	250	0.7
SnO_2_-ZnO aerogels [24]	300	0.01

## Data Availability

The data presented in this article are available on request from the corresponding author.
